# Supplementary material for: Pre-existing traits associated with Covid-19 illness severity
Source: PLoS One. 2020 Jul 23;15(7):e0236240. doi: 10.1371/journal.pone.0236240 (PMC7377468; doi:10.1371/journal.pone.0236240)
Supplement: S1 Table — (DOCX) [file pone.0236240.s001.docx]

**S1 Table. Elixhauser Comorbiditiy Index and Van Walraven Weights.**

| **Category** | **ICD-10 Codes**[24] | **van Walraven Weights**[23] |
| --- | --- | --- |
| Congestive heart failure | I09.9, I11.0, I13.0, I13.2, I25.5, I42.0, I42.5 - I42.9, I43.x, I50.x, P29.0 | 7 |
| Cardiac arrhythmias | I44.1 - I44.3, I45.6, I45.9, I47.x - I49.x, R00.0, R00.1, R00.8, T82.1, Z45.0, Z95.0 | 5 |
| Valvular disease | A52.0, I05.x - I08.x, I09.1, I09.8, I34.x - I39.x, Q23.0 - Q23.3, Z95.2 - Z95.4 | -1 |
| Pulmonary circulation disorders | I26.x, I27.x, I28.0, I28.8, I28.9 | 4 |
| Peripheral vascular disorders | I70.x, I71.x, I73.1, I73.8, I73.9, I77.1, I79.0, I79.2, K55.1, K55.8, K55.9, Z95.8, Z95.9 | 2 |
| Hypertension, uncomplicated | I10.x | 0 |
| Hypertension, complicated | I11.x - I13.x, I15.x | 0 |
| Paralysis | G04.1, G11.4, G80.1, G80.2, G81.x, G82.x, G83.0 - G83.4, G83.9 | 7 |
| Other neurological disorders | G10.x - G13.x, G20.x - G22.x, G25.4, G25.5, G31.2, G31.8, G31.9, G32.x, G35.x - G37.x, G40.x, G41.x, G93.1, G93.4, R47.0, R56.x | 6 |
| Chronic pulmonary disease | I27.8, I27.9, J40.x - J47.x, J60.x - J67.x, J68.4, J70.1, J70.3 | 3 |
| Diabetes mellitus, uncomplicated | E10.0, E10.1, E10.9, E11.0, E11.1, E11.9, E12.0, E12.1, E12.9, E13.0, E13.1, E13.9, E14.0, E14.1, E14.9 | 0 |
| Diabetes mellitus, complicated | E10.2 - E10.8, E11.2 - E11.8, E12.2 - E12.8, E13.2 - E13.8, E14.2 - E14.8 | 0 |
| Hypothyroidism | E00.x - E03.x, E89.0 | 0 |
| Renal failure | I12.0, I13.1, N18.x, N19.x, N25.0, Z49.0 - Z49.2, Z94.0, Z99.2 | 5 |
| Liver disease | B18.x, I85.x, I86.4, I98.2, K70.x, K71.1, K71.3 - K71.5, K71.7, K72.x - K74.x, K76.0, K76.2 - K76.9, Z94.4 | 11 |
| Peptic ulcer disease, excluding bleeding | K25.7, K25.9, K26.7, K26.9, K27.7, K27.9, K28.7, K28.9 | 0 |
| AIDS/HIV | B20.x - B22.x, B24.x | 0 |
| Lymphoma | C81.x - C85.x, C88.x, C96.x, C90.0, C90.2 | 9 |
| Metastatic cancer | C77.x - C80.x | 12 |
| Solid tumour without metastasis | C00.x - C26.x, C30.x - C34.x, C37.x - C41.x, C43.x, C45.x - C58.x, C60.x - C76.x, C97.x | 4 |
| Rheumatoid arthritis/collagen vascular diseases | L94.0, L94.1, L94.3, M05.x, M06.x, M08.x, M12.0, M12.3, M30.x, M31.0 - M31.3, M32.x - M35.x, M45.x, M46.1, M46.8, M46.9 | 0 |
| Coagulopathy | D65 - D68.x, D69.1, D69.3 - D69.6 | 3 |
| Obesity | E66.x | -4 |
| Weight loss | E40.x - E46.x, R63.4, R64 | 6 |
| Fluid and electrolyte disorders | E22.2, E86.x, E87.x | 5 |
| Blood loss anemia | D50.0 | -2 |
| Deficiency anemia | D50.8, D50.9, D51.x - D53.x | -2 |
| Alcohol abuse | F10, E52, G62.1, I42.6, K29.2, K70.0, K70.3, K70.9, T51.x, Z50.2, Z71.4, Z72.1 | 0 |
| Drug abuse | F11.x - F16.x, F18.x, F19.x, Z71.5, Z72.2 | -7 |
| Psychoses | F20.x, F22.x - F25.x, F28.x, F29.x, F30.2, F31.2, F31.5 | 0 |
| Depression | F20.4, F31.3 - F31.5, F32.x, F33.x, F34.1, F41.2, F43.2 | -3 |
