# Supplementary material for: Pre-existing traits associated with Covid-19 illness severity
Source: PLoS One. 2020 Jul 23;15(7):e0236240. doi: 10.1371/journal.pone.0236240 (PMC7377468; doi:10.1371/journal.pone.0236240)
Supplement: S2 Table — (DOCX) [file pone.0236240.s002.docx]

**S2 Table. Characteristics Associated with Need for Any Hospitalization in All Patients with Covid-19.**

|  | **Age- and Sex-Adjusted Models** | | **Multivariable-Adjusted Model*** | |
| --- | --- | --- | --- | --- |
|  | **OR (95% CI)** | ***P*** | **OR (95% CI)** | ***P*** |
| **Outcome: Severe Illness (N=214 needed any hospital admission, of the N=442 total diagnosed with COVID-19)** | | | | |
| **Age, per 10 years** | 1.88 (1.65,2.14) | <0.001 | **1.55 (1.32,1.81)** | **<0.001** |
| Male sex | 1.62 (1.04,2.52) | 0.034 | 1.64 (0.99,2.72) | 0.054 |
| African American race | 2.30 (1.18,4.50) | 0.015 | 1.66 (0.78,3.53) | 0.19 |
| Hispanic ethnicity | 1.65 (0.91,2.99) | 0.10 | 1.16 (0.58,2.33) | 0.67 |
| Obesity | 2.04 (1.14,3.65) | 0.016 | 1.99 (0.97,4.08) | 0.059 |
| Hypertension | 2.09 (1.27,3.44) | 0.004 | 1.14 (0.61,2.13) | 0.69 |
| **Diabetes mellitus** | 3.37 (1.82,6.24) | <0.001 | **2.81 (1.35,5.85)** | **0.006** |
| **Elixhauser comorbidity score, per SD** | 4.27 (2.64,6.90) | <0.001 | **4.34 (2.53,7.44)** | **<0.001** |
| Prior myocardial infarction or heart failure | 5.11 (1.71,15.28) | 0.004 | 0.69 (0.17,2.82) | 0.60 |
| Prior COPD or asthma | 1.53 (0.83,2.80) | 0.17 | 0.80 (0.38,1.65) | 0.54 |
| ACE inhibitor use | 0.79 (0.34,1.84) | 0.58 | 0.42 (0.14,1.25) | 0.12 |
| Angiotensin receptor blocker use | 0.90 (0.41,1.95) | 0.78 | 0.85 (0.34,2.09) | 0.72 |

*All listed covariates shown were included in the full multivariable-adjusted model.
