# Supplementary material for: Pre-existing traits associated with Covid-19 illness severity
Source: PLoS One. 2020 Jul 23;15(7):e0236240. doi: 10.1371/journal.pone.0236240 (PMC7377468; doi:10.1371/journal.pone.0236240)
Supplement: S3 Table — (DOCX) [file pone.0236240.s003.docx]

**S3 Table. Characteristics Associated with Distinct Outcomes in Patients Hospitalized for Covid-19.**

|  | **Age- and Sex-Adjusted Models** | | **Multivariable-Adjusted Model*** | |
| --- | --- | --- | --- | --- |
|  | **OR (95% CI)** | ***P*** | **OR (95% CI)** | ***P*** |
| **Outcome: Critical Illness (N=77 needing ICU level care, of N=214 admitted)** | | | | |
| Age, per 10 years | 1.14 (0.97,1.33) | 0.11 | 1.11 (0.93,1.32) | 0.25 |
| Male sex | 2.29 (1.23,4.26) | 0.009 | **2.41 (1.27,4.55)** | **0.007** |
| African American race | 1.75 (0.85,3.60) | 0.13 | 1.51 (0.72,3.18) | 0.28 |
| Hispanic ethnicity | 1.27 (0.58,2.78) | 0.55 |  |  |
| Obesity | 1.26 (0.62,2.57) | 0.52 | - | - |
| Hypertension | 1.56 (0.83,2.92) | 0.17 | 1.62 (0.84,3.11) | 0.15 |
| Diabetes mellitus | 1.18 (0.64,2.19) | 0.59 | - | - |
| Elixhauser comorbidity score, per SD | 1.12 (0.86,1.47) | 0.39 | - | - |
| Prior myocardial infarction or heart failure | 1.07 (0.52,2.23) | 0.85 | - | - |
| Prior COPD or asthma | 0.82 (0.40,1.68) | 0.59 | - | - |
| ACE inhibitor use | 0.43 (0.14,1.26) | 0.12 | 0.38 (0.13,1.17) | 0.09 |
| Angiotensin receptor blocker use | 1.47 (0.64,3.38) | 0.37 | - | - |
| **Outcome: Respiratory Failure (N=52 needing intubation, of N=214 admitted)** | | | | |
| Age, per 10 years | 1.15 (0.96,1.37) | 0.14 | 1.14 (0.95,1.38) | 0.18 |
| Male sex | 2.88 (1.36,6.06) | 0.005 | 2.99 (1.41,6.36) | 0.004 |
| African American race | 2.14 (0.99,4.64) | 0.053 | 2.14 (0.99,4.64) | 0.053 |
| Hispanic ethnicity | 1.35 (0.56,3.24) | 0.51 | - | - |
| Obesity | 1.57 (0.72,3.41) | 0.26 | - | - |
| Hypertension | 0.81 (0.40,1.64) | 0.57 | - | - |
| Diabetes mellitus | 0.94 (0.47,1.89) | 0.87 | - | - |
| Elixhauser comorbidity score, per SD | 0.86 (0.63,1.18) | 0.34 | - | - |
| Prior myocardial infarction or heart failure | 0.70 (0.30,1.64) | 0.41 | - | - |
| Prior COPD or asthma | 0.67 (0.29,1.53) | 0.34 | - | - |
| ACE inhibitor use | 0.57 (0.17,1.85) | 0.35 | - | - |
| Angiotensin receptor blocker use | 1.38 (0.56,3.42) | 0.48 | - | - |

*To avoid model overfitting given the sample size, covariates included in the multivariable model were selected from age- and sex-adjusted models based on significance with P<0.20.
