# Supplementary material for: Pre-existing traits associated with Covid-19 illness severity
Source: PLoS One. 2020 Jul 23;15(7):e0236240. doi: 10.1371/journal.pone.0236240 (PMC7377468; doi:10.1371/journal.pone.0236240)
Supplement: S4 Table — (DOCX) [file pone.0236240.s004.docx]

**S4 Table. Age, Sex, and Obesity interactions With Characteristics Associated with Overall Covid-19 Illness Severity in the Total Sample (N=442).**

|  | **Interaction with Age Group** | | **Interaction with Male Sex** | | **Interaction with Obesity** | |
| --- | --- | --- | --- | --- | --- | --- |
|  | **LRT Chi-square** | ***P value*** | **LRT Chi-square** | ***P value*** | **LRT Chi-square** | ***P value*** |
| Older age (≥52 years) | - | - | 1.31 | 0.25 | 10.97 | **<0.001** |
| Male sex | 1.31 | 0.25 | - | - | 0.01 | 0.94 |
| African American race | 3.95 | **0.047** | 3.38 | 0.07 | 0.30 | 0.59 |
| Hispanic ethnicity | 5.27 | **0.022** | 0.01 | 0.93 | 0.68 | 0.41 |
| Obesity | 10.97 | **<0.001** | 0.01 | 0.94 | - | - |
| Hypertension | 0.99 | 0.32 | 0.42 | 0.52 | 14.73 | **<0.001** |
| Diabetes mellitus | 3.96 | **0.047** | 0.38 | 0.54 | 3.23 | 0.07 |
| Elixhauser comorbidity score, per SD | 5.15 | **0.023** | 0.03 | 0.86 | 1.01 | 0.31 |
| Prior myocardial infarction or heart failure | 3.73 | 0.053 | 0.00 | 0.97 | 0.26 | 0.61 |
| Prior COPD or asthma | 1.86 | 0.17 | 0.35 | 0.56 | 3.40 | 0.07 |
| ACE inhibitor use | 0.97 | 0.33 | 0.50 | 0.48 | 0.97 | 0.32 |
| Angiotensin receptor blocker use | 0.02 | 0.90 | 0.22 | 0.64 | 1.97 | 0.16 |

*The primary outcome of Covid-19 illness severity score in the total sample was defined as an ordinal variable wherein: 0 = referent, 1 = required admission but never ICU level care, 2 = required ICU level care but never intubated, 3 = required intubation.
