# Supplementary material for: Pre-existing traits associated with Covid-19 illness severity
Source: PLoS One. 2020 Jul 23;15(7):e0236240. doi: 10.1371/journal.pone.0236240 (PMC7377468; doi:10.1371/journal.pone.0236240)
Supplement: S5 Table — (DOCX) [file pone.0236240.s005.docx]

**S5 Table. Age, Sex, and Obesity Stratified Associations with Overall Covid-19 Illness Severity in the Total Sample (N=442).**

|  | **Age** | **OR (95%CI)** | ***P*** | **Sex** | **OR (95%CI)** | ***P*** | **Obesity** | **OR (95%CI)** | ***P*** |
| --- | --- | --- | --- | --- | --- | --- | --- | --- | --- |
| **Older age (≥52 years)** | Age <52 | - | - | Women | 3.93 (2.00,7.69) | <0.001 | Non-obese | 4.07 (2.46,6.74) | <0.001 |
|  | Age ≥52 | - | - | Men | 2.44 (1.38,4.30) | 0.002 | Obese | 0.69 (0.26,1.82) | 0.45 |
| **Men** | Age <52 | 2.38 (1.26,4.49) | 0.008 | Women | - | - | Non-obese | 1.78 (1.15,2.75) | 0.01 |
|  | Age ≥52 | 1.48 (0.88,2.48) | 0.14 | Men | - | - | Obese | 1.86 (0.70,4.93) | 0.22 |
| **African American** | Age <52 | 0.78 (0.27,2.24) | 0.64 | Women | 1.07 (0.48,2.40) | 0.87 | Non-obese | 1.94 (1.03,3.68) | 0.042 |
|  | Age ≥52 | 2.66 (1.32,5.36) | 0.006 | Men | 2.96 (1.35,6.52) | 0.007 | Obese | 1.36 (0.43,4.33) | 0.60 |
| **Hispanic ethnicity** | Age <52 | 2.28 (1.08,4.79) | 0.03 | Women | 1.20 (0.52,2.78) | 0.67 | Non-obese | 1.40 (0.75,2.62) | 0.29 |
|  | Age ≥52 | 0.65 (0.29,1.42) | 0.28 | Men | 1.26 (0.61,2.62) | 0.53 | Obese | 0.81 (0.26,2.55) | 0.72 |
| **Obesity** | Age <52 | 4.84 (2.19,10.70) | <0.001 | Women | 1.75 (0.77,3.98) | 0.19 | Non-obese | - | - |
|  | Age ≥52 | 0.82 (0.40,1.70) | 0.60 | Men | 1.82 (0.88,3.79) | 0.11 | Obese | - | - |
| **Hypertension** | Age <52 | 1.97 (0.89,4.35) | 0.09 | Women | 1.23 (0.62,2.45) | 0.55 | Non-obese | 2.06 (1.21,3.52) | 0.008 |
|  | Age ≥52 | 1.24 (0.69,2.21) | 0.47 | Men | 1.61 (0.88,2.93) | 0.12 | Obese | 0.25 (0.09,0.70) | 0.008 |
| **Diabetes** | Age <52 | 3.70 (1.44,9.46) | 0.006 | Women | 1.40 (0.65,3.00) | 0.39 | Non-obese | 2.14 (1.17,3.91) | 0.013 |
|  | Age ≥52 | 1.20 (0.65,2.23) | 0.56 | Men | 1.88 (0.96,3.67) | 0.06 | Obese | 0.76 (0.28,2.08) | 0.60 |
| **Elixhauser index, per SD** | Age <52 | 3.59 (1.93,6.68) | <0.001 | Women | 1.93 (1.38,2.70) | <0.001 | Non-obese | 2.03 (1.57,2.64) | <0.001 |
|  | Age ≥52 | 1.80 (1.39,2.34) | <0.001 | Men | 1.99 (1.49,2.66) | <0.001 | Obese | 1.56 (0.93,2.60) | 0.09 |
| **Prior MI or heart failure** | Age <52 | 4.12 (0.56,30.52) | 0.17 | Women | 0.66 (0.24,1.82) | 0.42 | Non-obese | 0.61 (0.27,1.36) | 0.23 |
|  | Age ≥52 | 0.57 (0.27,1.20) | 0.14 | Men | 0.67 (0.29,1.55) | 0.35 | Obese | 0.86 (0.25,2.96) | 0.81 |
| **Prior COPD or asthma** | Age <52 | 1.26 (0.54,2.93) | 0.60 | Women | 0.62 (0.25,1.58) | 0.32 | Non-obese | 1.04 (0.56,1.91) | 0.91 |
|  | Age ≥52 | 0.59 (0.30,1.16) | 0.13 | Men | 0.87 (0.45,1.68) | 0.68 | Obese | 0.33 (0.12,0.96) | 0.042 |
| **ACEi use** | Age <52 | - | - | Women | 0.31 (0.08,1.22) | 0.09 | Non-obese | 0.58 (0.23,1.44) | 0.24 |
|  | Age ≥52 | 0.49 (0.22,1.08) | 0.08 | Men | 0.54 (0.22,1.35) | 0.19 | Obese | 0.26 (0.07,1.03) | 0.055 |
| **ARB use** | Age <52 | 1.11 (0.21,5.73) | 0.9 | Women | 1.00 (0.35,2.85) | 0.99 | Non-obese | 1.52 (0.73,3.15) | 0.26 |
|  | Age ≥52 | 1.24 (0.61,2.51) | 0.56 | Men | 1.36 (0.60,3.09) | 0.46 | Obese | 0.51 (0.13,2.03) | 0.34 |

*Patients were categorized by median age (i.e. 52 years), sex and obesity status. P for interactions were calculated from likelihood ratio test between models with and without the interaction term. For each variable in the list, age, sex, and obesity interaction terms are implemented in multivariable adjusted models, with other covariates representative of the entire cohort.
