# Supplementary material for: Pre-existing traits associated with Covid-19 illness severity
Source: PLoS One. 2020 Jul 23;15(7):e0236240. doi: 10.1371/journal.pone.0236240 (PMC7377468; doi:10.1371/journal.pone.0236240)
Supplement: S6 Table — (DOCX) [file pone.0236240.s006.docx]

**S6 Table. Characteristics Associated with Overall Covid-19 Illness Severity* in the Total Sample (N=442).**

|  | **Age- and Sex-Adjusted Models** | | **Multivariable-Adjusted Model**† | |
| --- | --- | --- | --- | --- |
|  | **OR (95% CI)** | ***P value*** | **OR (95% CI)** | ***P value*** |
| Age, per 10 years | 1.69 (1.52,1.88) | <0.001 | 1.48 (1.24,1.75) | <0.001 |
| Male sex | 1.90 (1.28,2.80) | 0.001 | 1.91 (1.14,3.18) | 0.014 |
| African American race∫ | 2.46 (1.45,4.18) | <0.001 | 1.87 (0.84,4.14) | 0.12 |
| Hispanic ethnicity | 1.54 (0.91,2.60) | 0.11 | 0.96 (0.45,2.05) | 0.92 |
| Obesity | 1.96 (1.19,3.24) | 0.009 | 1.48 (0.76,2.90) | 0.25 |
| Hypertension | 1.97 (1.27,3.05) | 0.003 | 1.02 (0.54,1.94) | 0.94 |
| Diabetes mellitus | 2.25 (1.41,3.57) | <0.001 | 2.43 (1.28,4.60) | 0.007 |
| Elixhauser comorbidity score, per SD | 1.63 (1.33,2.01) | <0.001 | 1.88 (1.39,2.54) | <0.001 |
| Prior myocardial infarction or heart failure | 1.72 (0.96,3.09) | 0.07 | 0.94 (0.41,2.17) | 0.89 |
| Prior COPD or asthma | 1.23 (0.75,2.03) | 0.41 | 0.69 (0.36,1.32) | 0.26 |
| ACE inhibitor use | 0.69 (0.35,1.38) | 0.29 | 0.41 (0.15,1.13) | 0.08 |
| Angiotensin receptor blocker use | 1.18 (0.63,2.19) | 0.61 | 0.87 (0.38,1.97) | 0.73 |
| Smoker | 0.30 (0.08,1.13) | 0.08 | 0.26 (0.06,1.11) | 0.07 |

*The primary outcome of Covid-19 illness severity score in the total sample was defined as an ordinal variable wherein: 0 = referent, 1 = required admission but never ICU level care, 2 = required ICU level care but never intubated, 3 = required intubation.

† All listed covariates shown were included in the full multivariable-adjusted model.

∫ Reference group is non-African American race
