# Supplementary material for: Pre-existing traits associated with Covid-19 illness severity
Source: PLoS One. 2020 Jul 23;15(7):e0236240. doi: 10.1371/journal.pone.0236240 (PMC7377468; doi:10.1371/journal.pone.0236240)
Supplement: S7 Table — (DOCX) [file pone.0236240.s007.docx]

**S7 Table. Characteristics Associated with Covid-19 Illness Severity Among All Hospitalized Patients.**

|  | **Age- and Sex-Adjusted Models** | | **Multivariable-Adjusted Model**† | |
| --- | --- | --- | --- | --- |
|  | **OR (95% CI)** | ***P value*** | **OR (95% CI)** | ***P value*** |
| Age, per 10 years | 1.13 (0.97,1.32) | 0.12 | 1.14 (0.92,1.41) | 0.22 |
| Male sex | 2.36 (1.29,4.34) | 0.006 | 2.88 (1.30,6.38) | 0.009 |
| African American race | 1.85 (0.92,3.71) | 0.08 | 2.76 (1.05,7.26) | 0.039 |
| Hispanic ethnicity | 1.31 (0.60,2.86) | 0.49 |  |  |
| Obesity | 1.32 (0.66,2.65) | 0.44 |  |  |
| Hypertension | 1.33 (0.72,2.46) | 0.37 |  |  |
| Diabetes mellitus | 1.15 (0.63,2.09) | 0.65 |  |  |
| Elixhauser comorbidity score, per SD | 1.04 (0.81,1.34) | 0.74 |  |  |
| Prior myocardial infarction or heart failure | 0.95 (0.47,1.94) | 0.89 |  |  |
| Prior COPD or asthma | 0.77 (0.38,1.54) | 0.46 |  |  |
| ACE inhibitor use | 0.45 (0.15,1.34) | 0.15 | 0.32 (0.07,1.53) | 0.15 |
| Angiotensin receptor blocker use | 1.45 (0.65,3.21) | 0.36 |  |  |
| Smoker | 0.00 (0.00,0.00) | <0.001 | 0.00 (0.00,0.00) | <0.001 |

*The secondary outcome of Covid-19 illness severity score in hospitalized patients was defined as an ordinal variable wherein: 1 = referents required admission but never ICU level care, 2 = required ICU level care but never intubate, 3 = required intubation.

†To avoid model overfitting given the sample size, covariates included in the multivariable model were selected from age- and sex-adjusted models based on significance with P<0.20
