# Supplementary figures and images for: Pre-existing traits associated with Covid-19 illness severity
Source: PLoS One. 2020 Jul 23;15(7):e0236240. doi: 10.1371/journal.pone.0236240 (PMC7377468; doi:10.1371/journal.pone.0236240)

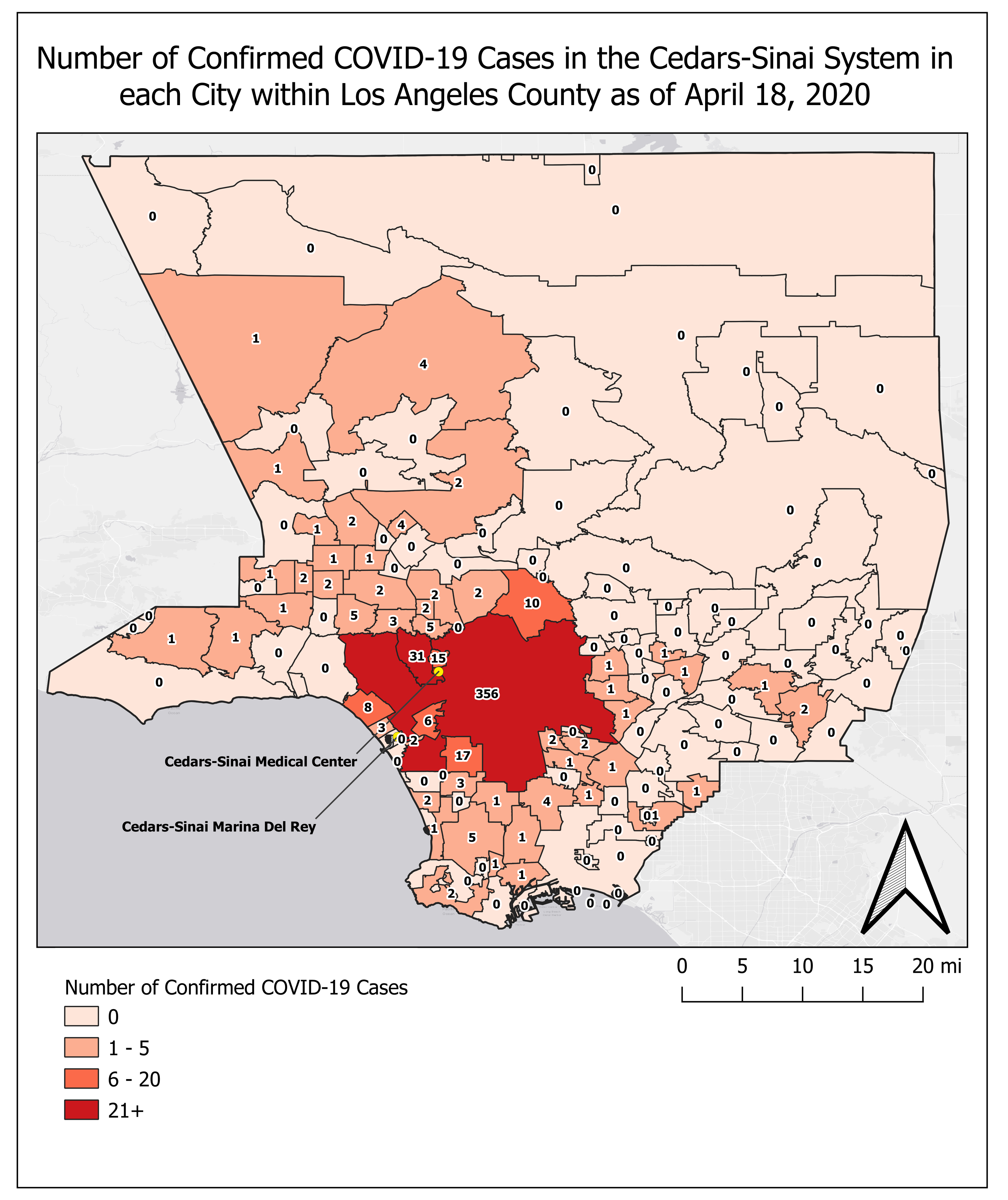

Supplement: S1 Fig — The patients treated in our healthcare system for Covid-19 illness presented from across a diverse regional distribution of residential locations across Los Angeles County. The map shown was generated using ArcGIS software by Esri. (TIF) [file pone.0236240.s008.tif]
